# Discontinuing Surveillance of Low‐Risk Branch‐Duct Intraductal Papillary Mucinous Neoplasms in Routine Practice: An External Application of the “Trivial” Criteria and the Resources it Would Release in Fourteen Health Systems

**DOI:** 10.1002/ueg2.70304

**Published:** 2026-10-09

**Authors:** Claudio Ricci, Vincenzo D'Ambra, Martina Casarotto, Laura Alberici, Carlo Ingaldi, Marco Fichera, Cristina Mosconi, Marina Migliori, Riccardo Casadei

**Affiliations:** ^1^ Department of Internal Medicine and Surgery (DIMEC) Alma Mater Studiorum University of Bologna Bologna Italy; ^2^ Division of Pancreatic Surgery IRCCS Azienda Ospedaliero‐Universitaria di Bologna Bologna Italy; ^3^ Division of Radiology IRCCS Azienda Ospedaliero‐Universitaria di Bologna Bologna Italy; ^4^ Division of Internal Medicine IRCCS Azienda Ospedaliero‐Universitaria di Bologna Bologna Italy

**Keywords:** branch‐duct intraductal papillary mucinous neoplasm, cost analysis, disinvestment, pancreatic cyst, surveillance

## Abstract

**Background:**

Surveillance of low‐risk branch‐duct intraductal papillary mucinous neoplasms is usually lifelong. Criteria to stop it after 5 years of stability in older patients have not been tested outside the center that devised them, nor has their economic value been measured.

**Objective:**

To test these criteria in an independent cohort and measure the lesions missed and the resources released.

**Methods:**

Single‐center cohort of patients with a low‐risk lesion at diagnosis was followed for at least 12 months with cross‐sectional imaging at baseline and during surveillance. The outcome was any new worrisome feature or high‐risk stigma. Risk after stability was estimated by conditional analysis. Released resources were valued at the marginal cost beyond the fifth year and priced with fourteen tariffs. Sensitivity analyses addressed discounting, horizon, adherence and basket composition.

**Results:**

Among 392 patients followed for 2375 person‐years, 66 (16.8%) developed a new feature; three had high‐grade dysplasia or carcinoma, and none died of the neoplasm. The 5‐year risk of a new feature did not fall with stability (14.3% after 2 years, 12.5% after five), but no resection and no high‐grade lesion occurred after 4 years. Discontinuation would have discharged 101 patients (25.8%), releasing 298 person‐years and 79,316 euro per 1000 patients entering surveillance, with no high‐grade lesion missed. Across health systems, the same rule released 42,000 to 170,000 euros.

**Conclusion:**

Discontinuation missed no high‐grade lesion, although the upper bound of that rate remained 0.84 per 100 person‐years. The resources released varied more than fourfold between health systems and must be recomputed locally.

## Key Summary

- Summarize the established knowledge on this subject
  ◦ Most branch‐duct intraductal papillary mucinous neoplasms without worrisome features behave indolently, yet surveillance is usually lifelong and requires substantial imaging.
  ◦ Criteria have been proposed to discontinue surveillance after 5 years of documented stability in patients aged 75 years or older with a cyst smaller than 30 mm, and in patients aged 65 years or older with a cyst 15 mm or smaller.
  ◦ Competing mortality is already an argument against prolonged surveillance in older patients, but the safety of these criteria has not been tested outside the center that proposed them, and what their application would release has never been measured.
- What are the significant and/or new findings of this study?
  ◦ Applied to an independent cohort of 392 patients, the published criteria would have discharged one quarter of them without missing a single high‐grade dysplasia or invasive carcinoma, although the exposure released is too small to exclude a rate of up to 0.84 lesions per 100 person‐years.
  ◦ The risk of a new worrisome feature does not decline with the duration of stability, but in this cohort its clinical consequence appeared to be no resection and no high‐grade lesion occurred after 4 years of documented stability, in a limited number of patients observed beyond that point.
  ◦ The resources released appear modest (€79,316 per 1000 patients entering surveillance) because surveillance costs are concentrated in the early years; valuing the released time at the average rather than the marginal cost overstates it by roughly three quarters.
  ◦ Pricing the same observed resource use with the tariffs of fourteen health systems changes the resources released more than fourfold, from about €42,000 to €170,000 per 1000 patients: the rule behaved safely in this cohort, but what it releases is a property of the health system that applies it.

## Graphical abstract

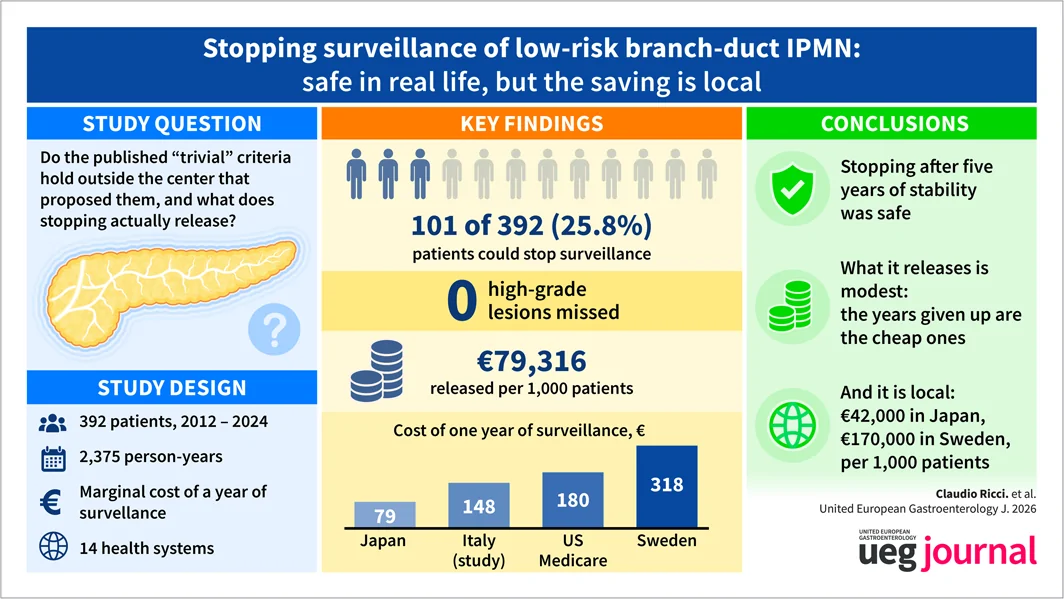

## 1 Introduction

Branch‐duct intraductal papillary mucinous neoplasms (BD‐IPMN) are discovered incidentally in a large and growing share of abdominal imaging studies, and most of them never progress [1, 2]. Because the minority that do progress cannot be identified reliably at diagnosis, guidelines recommend imaging surveillance; and in the absence of any agreed criterion for stopping, that surveillance is in practice lifelong [3, 4, 5]. The three major guidelines diverge precisely on this point, and none is founded on evidence that prolonged surveillance reduces mortality [6]. The workload falls on magnetic resonance capacity that is finite, and on patients who will, for the most part, never need an operation. Two distinct arguments support stopping. The first concerns the patient: in older people with comorbidity, competing causes of death remove any realistic prospect of benefit long before the lesion could become dangerous [7]. The second concerns the lesion: after a documented period without change, a cyst behaves so indolently that it may be regarded as trivial. On this reasoning, criteria have been proposed to discontinue surveillance after 5 years of stability in patients aged 75 years or older whose cyst has remained smaller than 30 mm, and in patients aged 65 years or older whose cyst has remained 15 mm or smaller [8, 9]. Neither argument, however, has been tested in an independent setting. The criteria were derived and validated within the center that proposed them, and their behavior in an independent cohort (with different referral patterns, a different operating threshold, and imperfect adherence in routine practice) is unknown. Less is still known about what discontinuation would release. Economic evaluations in this field are few and heterogeneous [10], and estimates of the cost of surveillance conventionally divide total expenditure by time, which assumes that a person‐year in the eighth year of follow‐up costs as much as a person‐year in the first; where examinations are concentrated early, that assumption inflates any saving attributed to stopping late. Because the price of an examination is set nationally or regionally, a figure from one health system cannot be carried into another. We therefore applied the published criteria to an independent cohort of patients under surveillance for a low‐risk branch‐duct neoplasm and asked two questions: how many high‐grade lesions the rule would have missed, and how much it would have released, here, and in thirteen other health systems.

## 2 Materials and Methods

### 2.1 Study Design and Population

We analyzed a prospectively maintained pancreatic cyst database of a single academic referral center. Patients entered the registry at the time of first diagnosis of a presumed BD‐IPMN and were followed according to the institutional protocol, which is based on the international consensus guidelines [3, 4]. For the current study, patients were eligible: (a) if the lesion carried neither a worrisome feature nor a high‐risk stigma at diagnosis; (b) if surveillance lasted at least 12 months, and if the work‐up was adequate (cross‐sectional imaging or endoscopic ultrasonography at baseline, and at least one magnetic resonance or computed tomography examination during follow‐up). Surveillance conducted with transabdominal ultrasonography alone cannot exclude progression, so those patients were excluded rather than counted as stable. The analysis covers patients diagnosed between 2012 and 2024.

### 2.2 Definitions and Outcomes

Worrisome features (WFs) and high‐risk stigmata (HRS) were defined according to the Kyoto 2024 consensus [3]. The primary outcome was the first new WFs or HRS, timed from diagnosis. Secondary outcomes were pancreatic resection, high‐grade dysplasia or invasive carcinoma on the resection specimen, and death from any cause or from the neoplasm.

The discontinuation rules examined were those proposed for lesions regarded as “trivial”: surveillance may stop in patients aged 75 years or older whose cyst has remained smaller than 30 mm, and in patients aged 65 years or older whose cyst has remained 15 mm or smaller, in both cases after 5 years free of WFs and HRS [8, 9]. Age was taken at the landmark. The two rules overlapped, and a third analysis applied whichever criterion the patient met.

### 2.3 Resource Use and Costing

Imaging and endoscopic procedures performed during surveillance were counted from the registry and valued at the tariffs of the regional schedule for outpatient specialist care, reported in full with their codes in Table S1. Endoscopic ultrasonography performed with tissue acquisition is billed under a single code that replaces, rather than adds to, the code for the diagnostic procedure. Transabdominal ultrasonography, even if performed at the patient's request, is not part of the surveillance protocol and, where it was obtained, was disregarded in both the resource counts and the costing. Computed tomography was costed whenever it replaced magnetic resonance, either because of a contraindication or because it was already being obtained for the oncological follow‐up of a previous malignancy. Each cross‐sectional examination is performed in the context of one specialist review, with a serum CA 19‐9 determination requested alongside it; the registry does not hold a count of either, so both were entered in the same number as the cross‐sectional examinations and priced at codes 89.01.9 and 90.55.3 of the same regional schedule. The baseline work‐up, patient time and travel were not costed, so the figures remain an underestimate of the total cost of surveillance. Tariffs are undiscounted and expressed in current euros. The economic analysis is reported according to the CHEERS 2022 statement [11].

Because examinations are concentrated in the early years, the cost of an additional year of surveillance is not the average cost per person‐year. We therefore estimated the marginal cost by regressing each patient's observed cost on surveillance duration among those followed beyond 5 years; the slope is the cost of one additional person‐year, and the same regression applied to procedure counts gives the marginal number of examinations. Resources released by discontinuation were valued at this marginal cost, assuming that surveillance would otherwise have continued to 10 years from diagnosis or to the end of observed follow‐up, whichever came first. Ten years was chosen so that the estimate remains within the period the cohort actually contributes; beyond it the estimate would be extrapolation rather than measurement, and in a cohort with a median age of 69 years it would be governed mainly by assumptions about competing mortality. The horizon was varied between seven and 20 years, costs were discounted at 0%, 3%, 3.5% and 5% per annum to the landmark at 5 years, and the composition of the basket was varied in sensitivity analysis (Table S5). The average cost was retained as a sensitivity analysis.

### 2.4 Valuation Across Health Systems

To assess whether the estimate transfers, we priced the resource use observed in this cohort (expressed as magnetic resonance and computed tomography examinations per person‐year) using published tariffs from fourteen health systems. We excluded endoscopic ultrasonography from this comparison because it has no comparable code across systems, and the specialist review and the CA 19‐9 determination because their tariffs are not published in a comparable form; the comparison therefore prices cross‐sectional imaging only. A separately priced diagnostic biliopancreatic examination could be identified in 10 of the fourteen schedules and is absent from five; where it exists the amounts are not commensurable, some being professional fees with the facility cost met separately, others all‐in episode prices, and in one system a surcharge on the base endoscopy (Table S5). Because actual‐to‐intended imaging ratios are not published for the other health systems, the resources released are reported for each system both at the adherence observed here and under full protocol adherence. Tariffs were taken from national or regional fee schedules and, where a payment is split between a professional and a technical component, both were included; values in other currencies were converted at the European Central Bank reference rates of 21 August 2026. Every source, and every health system for which no comparable unit price could be established, is listed in Table S2.

### 2.5 Statistical Analysis

Freedom from a new WFs or HRS was estimated using the Kaplan–Meier method. The risk conditional on a documented period without change was obtained as one minus the ratio of the survival function at the landmark plus 60 months to its value at the landmark, and confirmed by landmark analysis with time reset at that point (Table S4); the two are algebraically equivalent, and their agreement verifies the computation rather than adding information. Confidence intervals for conditional risks and for the marginal cost were obtained by bootstrap resampling with 2000 replicates. The rate of undetected high‐grade lesions was estimated from the person‐years contributed by the patients each rule would have discharged. Because a rate estimated from few or no events cannot be summarized by a point estimate alone, we derived its whole distribution with a Bayesian method suited to rare events (a Jeffreys prior on a Poisson rate), which yields both the expected number of lesions that would go undetected and the probability that there would be none. A Monte Carlo model with 50,000 simulations then propagated, jointly, the proportion of patients eligible for discontinuation, the person‐years released for each, the marginal cost per person‐year and that rate, yielding the joint distribution of resources released and lesions not detected. Continuous variables are reported as median and interquartile range, categorical variables as counts and percentages. The prior, the resulting posterior and every input distribution of the Monte Carlo model are set out in full in Table S5. Analyses were performed in Python 3.11 and R 4.3.3.

## 3 Results

### 3.1 Cohort

Of 777 patients in the registry, 392 met the study criteria (Figure 1). Table 1 gives the full description. The most common reason for exclusion was the presence of a WF or HRS at diagnosis in 158 patients; of the 498 patients with an adequate baseline work‐up, 52 were excluded because the record of the procedures performed had not been compiled and the modality of surveillance could not be established, and a further 54 because surveillance was conducted with transabdominal ultrasonography alone. The cohort was elderly and comorbid: median age 69 years (interquartile range 60–74), 319 patients (81.4%) with at least one comorbidity, 131 (33.4%) with a previous malignancy. The cysts were small, with a median size of 12 mm (8–17), with 242 (61.7%) below 15 mm, and multifocal in 219 patients (55.9%). Baseline work‐up included magnetic resonance in 353 patients (90.1%). Surveillance lasted a median of 67 months (40–97), for a total of 2375 person‐years, and comprised a median of three cross‐sectional or endoscopic procedures per patient (2–5).

**FIGURE 1:**
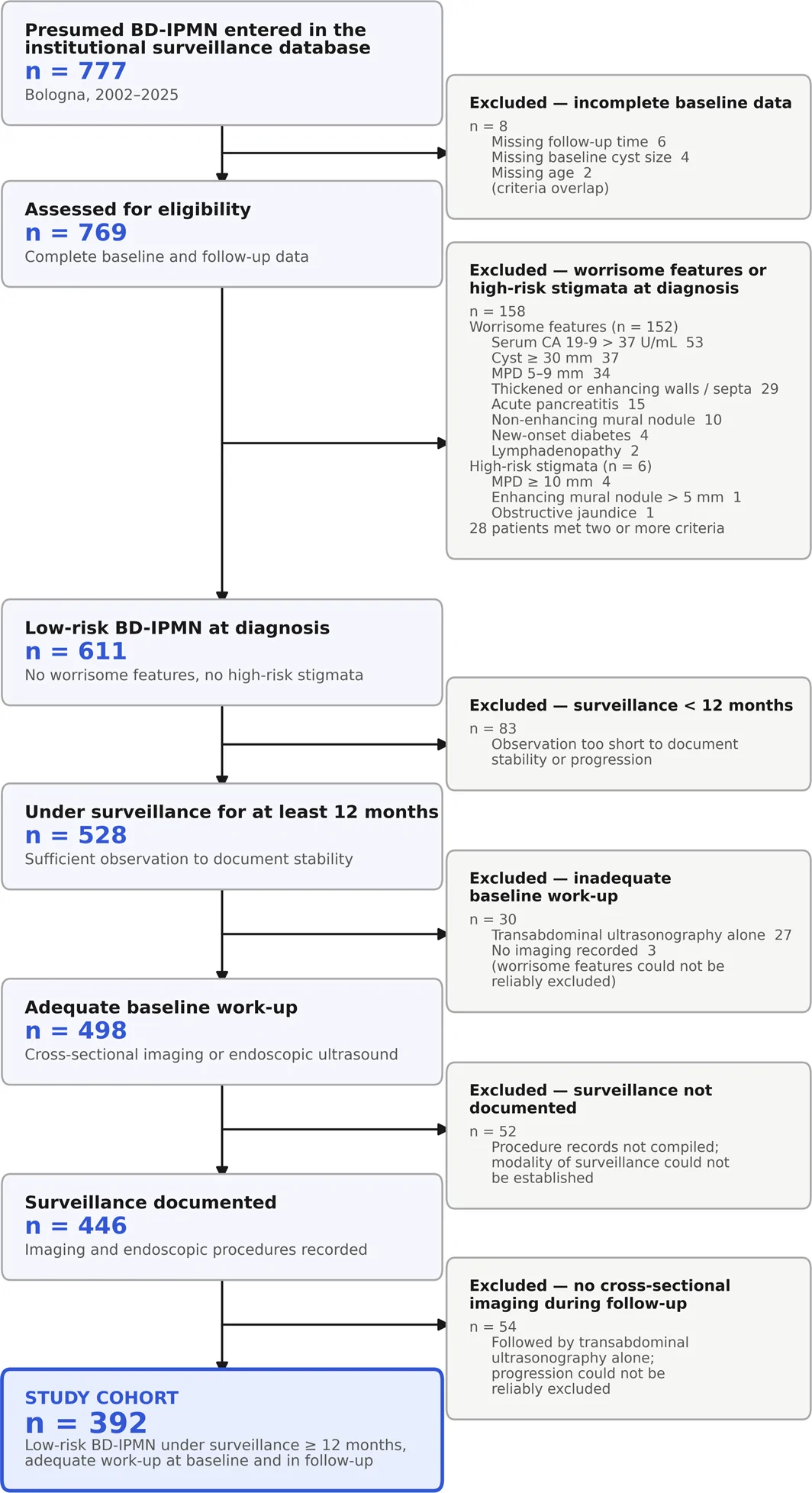
Selection of the study cohort. Flow of patients from the institutional pancreatic cyst registry to the analyzed cohort. Worrisome features and high‐risk stigmata were defined according to the Kyoto 2024 international consensus guidelines; the counts of individual criteria are not mutually exclusive. Adequate work‐up required cross‐sectional imaging or endoscopic ultrasonography at baseline and at least one magnetic resonance or computed tomography examination during follow‐up. BD‐IPMN = branch‐duct intraductal papillary mucinous neoplasm; MPD = main pancreatic duct.

**TABLE 1:** Characteristics of the study cohort, surveillance delivered and outcomes.

Characteristic	Study cohort (*n* = 392)
Patient characteristics at diagnosis	
Age, years	69 (60–74)
Female sex	257 (65.6)
Charlson comorbidity index	3 (2–5)
Any comorbidity	319 (81.4)
Family history of pancreatic cancer	20 (5.1)
Previous pancreatitis	5 (1.3)
Previous malignancy	131 (33.4)
Immunosuppression	16 (4.1)
Cyst characteristics at diagnosis	
Largest cyst diameter, mm	12 (8–17)
Cyst < 15 mm	242 (61.7)
Cyst 15–29 mm	150 (38.3)
Multifocal disease	219 (55.9)
Head involved	274 (69.9)
Body involved	243 (62.0)
Tail involved	169 (43.1)
Main pancreatic duct diameter, mm	3.0 (3.0–3.0)
Serum CA 19–9, U/mL	8.6 (4.6–15.9)
Baseline diagnostic work‐up	
MRI alone	296 (75.5)
MRI plus EUS	57 (14.5)
CT scan alone	34 (8.7)
CT scan plus EUS	5 (1.3)
Surveillance delivered	
Duration of surveillance, months	67 (40–97)
Imaging and endoscopic procedures per patient, *n*	3 (2–5)
Fine‐needle aspiration or biopsy during surveillance	25 (6.4)
Procedures expected per guidelines, *n* ^a^	4.8 (3.1–7.4)
Ratio of procedures performed to expected^a^	0.65 (0.43–1.00)
Under‐surveilled for interval	234 (59.7)
Cost of surveillance	
Total cost per patient, €^b^	853 (569–1422)
Cost per person‐year, €^b^	185 (95–280)
Outcomes during surveillance	
New WFs or HRS^c^	66 (16.8)
Cyst growth ≥ 2.5 mm/year	47 (12.0)
Thickened or enhancing walls/septa	18 (4.6)
Cyst ≥ 30 mm	7 (1.8)
Serum CA 19‐9 > 37 U/mL	3 (0.8)
Mural nodule	6 (1.5)
Main pancreatic duct ≥ 5 mm	1 (0.3)
Surgical resection	6 (1.5)
High‐grade dysplasia or invasive carcinoma	3 (0.8)
Death from any cause	36 (9.2)
Death from IPMN	0 (0.0)

*Note:* Continuous variables are reported as median (interquartile range), and categorical variables as number (%).

Abbreviations: CT = Computed Tomography; EUS = Endoscopic Ultrasound; HRS= High Risk Stigmata; MRI = Magnetic Resonance Imaging; WFs = worrisome features.

^a^ Number expected under the institutional protocol, and the ratio of those performed to those expected.

^b^ Valued at the regional tariffs listed in Table S1.

^c^ A new worrisome feature or high‐risk stigma appearing during surveillance in a patient free of that feature at diagnosis; categories are not mutually exclusive. CA 19‐9, carbohydrate antigen 19‐9.

Only two‐thirds of the cross‐sectional examinations the protocol prescribes were performed: 0.64 per person‐year versus 0.95 expected, a ratio of 0.67.

### 3.2 Progression During Surveillance

Sixty‐six patients (16.8%) developed a new worrisome feature or high‐risk stigma. Freedom from such an event was 95.6% at 2 years (95% confidence interval 93.6–97.7), 87.3% at 5 years (83.7–91.0) and 76.4% at 10 years (70.2–82.7) (Figure 2). Six patients (1.5%) underwent resection, and three (0.8%) proved to have high‐grade dysplasia or invasive carcinoma. Thirty‐six patients died, none from the neoplasm.

**FIGURE 2:**
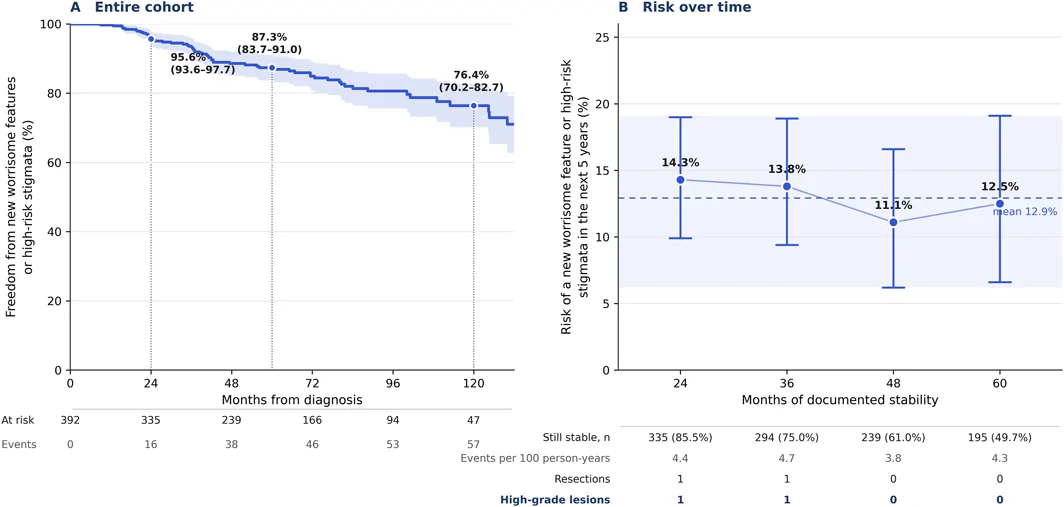
Progression during surveillance and risk after a documented period of stability. (A) Kaplan–Meier estimate of freedom from a new worrisome feature or high‐risk stigma, with the numbers at risk and the cumulative events below the axis. (B) Probability of a new worrisome feature or high‐risk stigma within the following 5 years, according to the duration of stability already documented, with 95% confidence intervals obtained by bootstrap resampling. The rows beneath give, for each landmark, the number of patients still free of events, the incidence rate, and the numbers who subsequently underwent resection or proved to harbor high‐grade dysplasia or invasive carcinoma.

### 3.3 Risk After a Documented Period of Stability

The risk of a new feature within the following 5 years did not decline as stability lengthened: 14.3% (9.9–19.0) after 2 years without change, 13.8% (9.4–18.9) after three, 11.1% (6.2–16.6) after four and 12.5% (6.6–19.1) after five. The incidence rate was likewise stable, between 3.8 and 4.7 events per 100 person‐years at every landmark. Only one patient stable beyond 2 years was later operated on, after almost 4 years without change, and the specimen showed high‐grade dysplasia. No patient stable for 4 years or more underwent resection or had a high‐grade lesion (Figure 2).

### 3.4 Applying the Discontinuation Rules

One hundred and ninety‐five patients (49.7%) reached 5 years free of WFs and HRS. Of these, 71 met the criterion of age 75 years or more with a cyst below 30 mm, 77 met the criterion of age 65 years or more with a cyst of 15 mm or less, and 101 met one or the other, representing 25.8% of all patients entering surveillance (Table 2).

**TABLE 2:** Resources released and clinical consequences of applying published discontinuation rules to a real‐world cohort.

Discontinuation rule applied after 5 years of stability^a^	Age ≥ 75 years, stable cyst < 30 mm	Age ≥ 65 years, stable cyst ≤ 15 mm	Either rule
Resources released			
Patients eligible for discontinuation, n	71	77	101
% of the study cohort	18.1	19.6	25.8
% of patients stable at 5 years	36.4	39.5	51.8
Person‐years of surveillance avoided	195	234	298
Imaging and endoscopic procedures avoided, *n*	73	88	113
Cost avoided, €^b^	20,309	24,377	31,092
95% confidence interval	10,745–30,682	12,897–36,827	16,450–46 972
Cost avoided per 1000 patients stable at 5 years, €^c^	104,149	125,010	159,446
Cost avoided per 1000 patients entering surveillance, €^c^	51,809	62,186	79,316
Clinical consequences			
New WFs or HRS not detected, *n* ^d^	5	3	5
per 100 person‐years	2.6	1.3	1.7
Pancreatic resections not performed, *n*	0	0	0
High‐grade dysplasia or invasive carcinoma missed, *n*	0	0	0
Upper limit for missed lesions, per 100 person‐years^e^	1.29	1.07	0.84

Abbreviations: CI = confidence interval; HRS = High Risk Stigmata; WFs = Worrisome Features.

^a^ Age taken at the landmark. The two rules overlap; the third column applies whichever criterion the patient met.

^b^ Valued at the marginal cost of surveillance beyond the fifth year, €104.25 per person‐year (95% CI 55–157), with surveillance assumed to continue to 10 years from diagnosis or to the end of observed follow‐up.

^c^ Fewer than half of the patients entering surveillance reach 5 years of stability, so the two denominators differ; the second is the one relevant to a health service.

^d^ Events occurring after the landmark in patients who would have left surveillance.

^e^ Upper bound of the 95% credible interval for the rate of high‐grade lesions, from a Jeffreys posterior on the observed exposure.

Discharging those 101 patients would have released 298 person‐years of surveillance and left five new WFs undetected, an incidence of 1.7 per 100 person‐years among the discharged. No resection and no high‐grade dysplasia or invasive carcinoma would have been missed under either rule taken alone or under the two combined, although with 298 person‐years of released exposure the upper 95% bound of that rate is 0.84 per 100 person‐years, which corresponds to as many as two or three lesions that could have gone undetected.

### 3.5 What Discontinuation Releases

Cost did not accumulate in proportion to time. Among patients followed beyond 5 years, each additional year of surveillance cost 104 euro (95% confidence interval 55–157) and comprised 0.38 examinations, against an average of 180 euro per person‐year over the whole period. The average therefore overstates the cost of an additional year by roughly three quarters. Valued at this marginal cost, the 298 person‐years released amount to 31,092 (16,450–46,972) euro in this cohort, or 79,316 euro per 1000 patients entering surveillance. The two rules taken separately released 51,809 and 62,186 euro per 1000. Imaging and endoscopy alone, without the accompanying review and CA 19‐9, give 91 euro per person‐year and 69,523 euro per 1000 (Table S5).

### 3.6 Variation Across Health Systems

The observed pattern of resource use, 0.54 magnetic resonance and 0.10 computed tomography examinations per person‐year, cost 148 euro per person‐year at the tariffs of the study setting. The same pattern cost 79 euro in Japan and 318 euro when priced with the list prices of a Swedish regional schedule, a range of fourfold; restricting the comparison to the health systems for which a complete public payer tariff could be verified still left a range from 79 to 180 euro, a factor of 2.3 (Figure 3). Expressed as the resources released by the same discontinuation rule, the resources released ranged from 42,000 to 170,000 euro per 1000 patients entering surveillance at the adherence observed here, and from 101,000 to 406,000 euro if every protocol‐intended examination was performed (Table S5).

**FIGURE 3:**
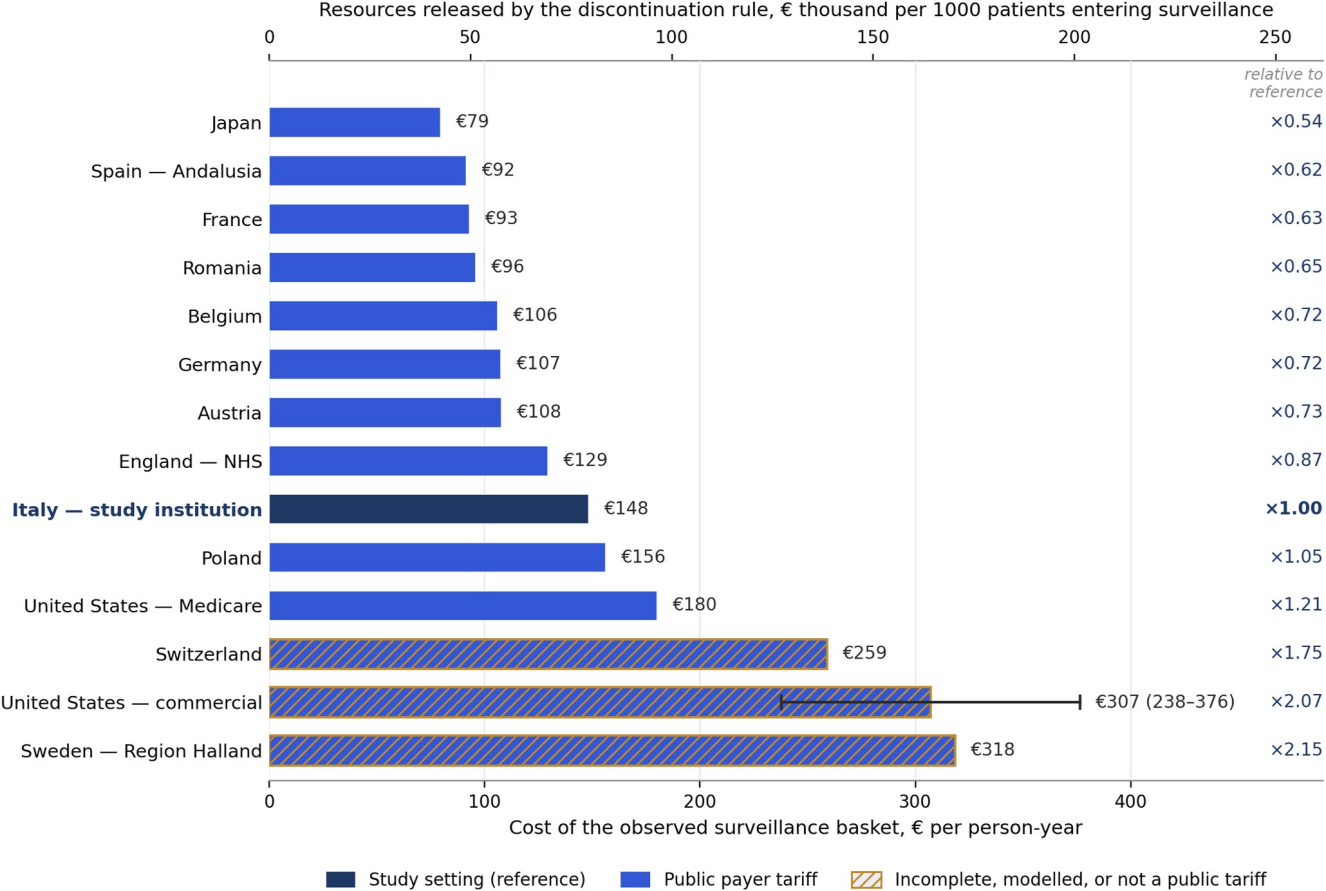
Cost of the observed surveillance across fourteen health systems. The resource use observed in the study cohort was 0.54 magnetic resonance and 0.10 computed tomography examinations per person‐year, priced with the tariffs of each health system. The lower axis gives the cost of one person‐year of surveillance; the upper axis converts it into the resources released by the combined discontinuation rule per 1000 patients entering surveillance. Values in other currencies were converted at the European central bank reference rates of 21 August 2026. Hatching marks the systems whose figures are not a complete public payer tariff; every source is given in Table S2.

### 3.7 Uncertainty and Sensitivity

We repeated the calculation 50,000 times, each time drawing a plausible value for every uncertain quantity: how many patients would be discharged, for how long, what a further year of surveillance would cost, and how often a high‐grade lesion would go undetected. Each simulation gives one point in Figure 4, panel A, placed by the money released and by the lesions missed. Half of the simulated cohorts released more than 79,286 euro per 1000 patients entering surveillance, and 95% fell between 42,392 and 125,162 euros, with the middle of the cloud matching the figure obtained by direct calculation. No lesion escaped detection in 54% of simulations and at most one in 73%; the expected number was 1.3 per 1000, a rate of 0.07 per 100 person‐years (0–0.84). Observing no lesions therefore indicates that the rate is low, not that no lesion would ever be missed. Two things move that answer, and both act on the same axis: what an examination costs, and how much surveillance is delivered. Figure 4, panel B maps them together, our cohort sitting at 104 euro per person‐year and 25.8% eligible. Duration of stability mattered most: halving it from 5 years to three raised the resources released to 144,142 euro per 1000, and requiring seven years reduced them to 41,816, with no high‐grade lesion missed at any landmark. Costing the released years at the average raised the estimate to 136,972 euro per 1000, and at the rate the protocol expects the rule would have released 189,268 euro per 1000 (Table S3). Discounting at 3% reduced the estimate to 74,985 euro and extending the horizon to the end of observed follow‐up raised it to 89,150 euro (Table S5).

**FIGURE 4:**
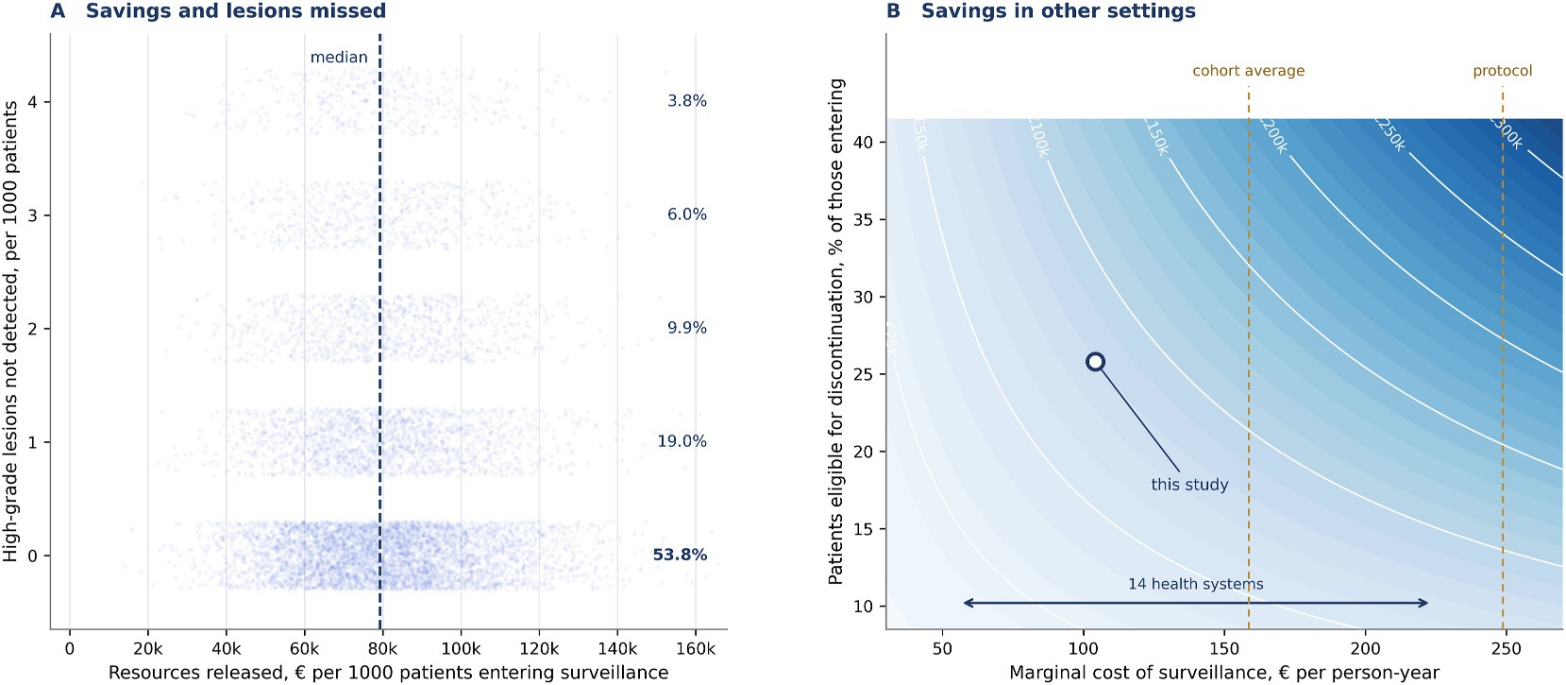
Probabilistic analysis and the determinants of the resources released. (A) Joint distribution of the resources released and of the high‐grade lesions that would not have been detected, from 50,000 simulations propagating the uncertainty in eligibility, in the person‐years released, in the marginal cost per person‐year and in the rate of undetected lesions. Points are jittered vertically for legibility and the percentages give the proportion of simulations at each count. The dashed line marks the median of the simulations. (B) Resources released as a function of the marginal cost of surveillance and of the proportion of patients eligible for discontinuation, with the study cohort marked. The horizontal arrow spans the fourteen health systems in Figure 3, and the vertical lines mark what the same rule would release if surveillance beyond the fifth year continued at the average rate of this cohort or at the rate the protocol expects.

## 4 Discussion

Applied to an independent cohort, the published criteria for discontinuing surveillance of a trivial BD‐IPMN would have discharged a quarter of patients entering surveillance without missing a single high‐grade dysplasia or invasive carcinoma. What that discharge would have released, however, was modest, about 79,300 euro per 1000 patients entering surveillance, and the same rule applied to the same patients released between 42,000 and 170,000 euro depending on whose tariffs were used to value it. In other words, the clinical case for discontinuation may transfer from one health system to another in a way that the economic case does not. The observation with the clearest clinical implication is a dissociation between the risk of a new event and the consequences of that event, and we set it out with the caution its sample size requires. The probability that a new WF will appear within 5 years is essentially unchanged whether a cyst has been stable for 2 years or for 5 years, and the incidence rate hovers around four events per 100 person‐years at every landmark. The reassurance that clinicians give, and that patients receive, when a cyst has been stable for 5 years is therefore not obviously supported by the behavior of the lesion in these data. What does change is the meaning of those events. Beyond 4 years of documented stability, the worrisome features that appeared were not followed by resection or by a high‐grade lesion in this cohort. The number of patients observed beyond that point is limited, and the finding should be read as a signal that the consequences of a new feature may attenuate with the duration of stability, not as evidence that they disappear. The distinction matters because a worrisome feature is a trigger for closer assessment and not a diagnosis: applied prospectively, the Kyoto criteria identify malignancy with a positive predictive value of about one in five, so most patients in whom a feature appears do not harbor a high‐grade lesion [12]. Others have reported malignant progression well beyond the fifth year (5.5% in a referral series and a cumulative incidence that continues to rise in a large Japanese cohort [13, 14]), while a community‐based cohort of cysts unchanged during the first 5 years found none [15]. That the appearance of a new worrisome feature carries prognostic weight early in surveillance [16] but appeared to carry less weight after 4 years of stability has not, to our knowledge, been reported before; it rests on a small number of patients observed beyond that point and needs confirmation. This is consistent with the excellent disease‐specific survival reported when worrisome features are managed conservatively [17]. This nuances the concept of a trivial cyst rather than confirming or refuting it. In our opinion, a cyst should be considered trivial not when it does not change, but when the changes it shows no longer modify its management.

Our findings should be read as an external application of criteria devised elsewhere, and the comparison must be made honestly. In the cohort in which those criteria were developed, 24 of 1617 patients still under surveillance beyond 5 years were found to harbor high‐grade dysplasia or invasive carcinoma, a rate of 1.5% [9]. Applied to the 101 patients our rule would have discharged, that rate predicts 1.5 such lesions, and the probability of observing none, if it were the true rate, is 22%. Our zero is therefore entirely compatible with their estimate and does not contradict it; it reflects a smaller denominator, a shorter horizon and a resection rate of 1.5% against their 4.3%. The upper bound of our own posterior distribution, 0.84 lesions per 100 person‐years, corresponds to as many as two or three missed lesions across the person‐years released. The rule was not seen to fail in our hands, but the confidence interval around that statement is wide, and a single‐center cohort of this size cannot narrow it; we therefore describe our result as compatible with safety rather than as evidence of it. An international cohort of more than three thousand patients has suggested that surveillance may be stopped in cysts below 20 mm that have not changed within 5 years [18], and an Italian center has recently reported the clinical outcomes of guideline‐based discontinuation in routine practice [19]. What those studies do not provide, and what this one attempts to add, is the value of the resources that discontinuation releases, and the finding that this value depends less on the rule than on the health system that applies it.

The two arguments that already exist for stopping surveillance refer to two different aspects. One concerns the patient and holds that competing mortality removes the prospect of benefit in the elderly [7]; the other concerns the lesion and holds that a cyst which has not changed will not change [8, 9]. This study deals with a third aspect, surveillance itself, and asks what it costs and whether that cost means the same thing anywhere else. The answer to the second question is no, for reasons that deserve comment. The cost of surveillance is not proportional to its duration. Examinations cluster in the first years, when the lesion is being characterized, and thin out thereafter; the marginal cost of a further year beyond the fifth was 104 euro against an average of 180 euro over the whole period. Any analysis that values released time at the average cost therefore inflates the saving in our data by about three quarters. This problem is not limited to pancreatic cysts. Disinvestment always removes the tail of a program, and in most surveillance programs, the tail is the least expensive part. Valuing it at the average cost is therefore a systematic error, and one that favors every proposal to stop surveillance late.

This has an uncomfortable implication: if the aim is to reduce the cost of surveillance, the elderly patient in the sixth year is not the right target because most of the expenditure is concentrated in the first 2 years and depends on the imaging modality chosen. The levers that matter are therefore upstream of discontinuation. The first is entry: a proportion of the cysts that enter surveillance are small, unifocal and found incidentally in elderly patients in whom competing mortality is already decisive, and a more selective entry policy would remove the expensive early years rather than the cheap late ones. The second is intensity: lengthening the interval between examinations in patients at the lowest risk captures part of the same resource without discharging anyone, and parametric models allow that interval to be set by age and cyst size rather than by a fixed protocol [20].

Two further findings bear on transferability. First, unit prices differ enormously: the identical pattern of examinations costs 79 euro per person‐year in Japan and 180 euro under United States Medicare, and higher still where prices are not set by a public payer. Savings computed in one health system cannot be quoted in another, and guidelines that attach economic claims to clinical recommendations should state which prices they used. Second, and less obvious, the intensity with which surveillance is actually delivered acts on the same quantity as the tariff. Our patients received only 67% of the cross‐sectional examinations the protocol expects (0.64 per person‐year against 0.95), the signature of constrained magnetic resonance capacity and imperfect attendance rather than a deliberate protocol. Had surveillance beyond the fifth year continued at the rate the protocol expects, the same rule would have released more than twice as much. The modest figure we report is therefore not evidence that the rule is ineffective; it may instead reflect how little late surveillance is actually being delivered. When the patient pays directly, the arithmetic changes completely. At commercial US rates, the observed pattern of surveillance costs about 307 euro per person‐year, and a decade of it approaches 3100 euro before any consideration of deductibles and co‐insurance. In such a setting, the case for discontinuing surveillance of a lesion that has been stable for 5 years ceases to be an argument about health service budgets and becomes an argument about the financial burden borne by the individual patient.

This study has clear limitations. First, it is retrospective and single‐center, and although the registry is prospectively maintained, the intensity of surveillance reflects local practice and local capacity. The number of patients the rule would discharge is small: 101 patients contributing 298 person‐years. The analysis is not powered to exclude a clinically relevant rate of undetected high‐grade lesions, and with no event observed the upper 95% bound of the posterior rate is 0.84 per 100 person‐years, which corresponds to as many as two or three lesions that could have gone undetected across the person‐years released. Our data are therefore compatible with the safety of the rule but do not establish it, and they are equally compatible with the 1.5% rate reported in the cohort in which the criteria were derived. The cohort is elderly by construction because the criteria apply only to patients aged 65 years or older; this is the population in which the rule is intended to operate rather than a feature of our referral pattern, but it also means that the findings say nothing about younger patients. To minimize this bias, we excluded patients without a cross‐sectional examination, a selection made on information accruing after baseline. Moreover, a third of the cross‐sectional examinations the protocol expects were not performed, which may have reduced the sensitivity with which events were detected and could in principle have contributed to the absence of high‐grade lesions after 4 years of stability, although no patient died of the neoplasm. Nonetheless, the cohort reflects surveillance as it is actually delivered, which is the setting in which the criteria would be applied.

What we report is the resources that discontinuation would release, not the net economic or health benefit of discontinuing. Two downstream consequences are left unmodeled and they pull in opposite directions: a lesion that escapes detection may present later at a more advanced stage, while continued surveillance generates operations for worrisome features that prove not to be malignant. A full cost‐effectiveness model would have to represent both, together with the stage distribution at presentation and the operative mortality of pancreatic resection, and would rest on assumptions that our data cannot inform.

A second limitation was that the costing takes the perspective of the payer and omits outpatient visits, laboratory tests, patient time, travel and the psychological burden of remaining under surveillance, all of which would increase the value of discontinuation, although the evidence on that burden is itself contested, one prospective study describing a Sword of Damocles effect [21] and another finding the psychological cost of cyst surveillance to be low [22]. Cost is borne by the patient as well as by the payer, and financial toxicity has already been documented in this population [23]. The tariff comparison, finally, is complete for the health systems reported but could not be established for several others, including much of northern Europe, and internal Italian variation could not be quantified.

Three further caveats attach to the economic component. The cross‐system comparison prices cross‐sectional imaging only, and assumes that the pattern of resource use observed here would be reproduced elsewhere, which adherence data from other systems would be needed to test. The specialist reviews and CA 19‐9 determinations entered in the base case were derived from the structure of the protocol rather than counted in the registry. Endoscopic ultrasonography is performed on indication rather than on a fixed schedule, so its contribution to the cost of surveillance is concentrated in patients whose lesion changed or had already been assessed endoscopically at diagnosis. None of these alters the direction of our conclusions, but each widens the uncertainty around their magnitude.

Within these limits, three conclusions follow. Discontinuing surveillance of a low‐risk branch‐duct neoplasm after 5 years of documented stability was compatible with safety in this cohort, with no high‐grade lesion observed, although the exposure released cannot exclude a rate of up to 0.84 per 100 person‐years. The resources released appear modest, and smaller than the literature would suggest, because the years being given up are the inexpensive ones. The size of that release appears to be a local quantity, determined by local prices and by how much surveillance a health system can actually deliver, and it should therefore be recalculated locally rather than taken from other settings.

## Funding

The authors have nothing to report.

## Ethics Statement

The Comitato Etico di Area Vasta Emilia Centro (PANBO 064/2017/U/Oss) approved this retrospective analysis of prospectively collected data and conducted it in accordance with the Declaration of Helsinki.

## Consent

All patients gave written informed consent for the use of their clinical data for research at the time of enrollment in the institutional pancreatic cyst surveillance program.

## Conflicts of Interest

The authors declare no conflicts of interest.

## Permission to Reproduce Material From Other Sources

The authors have nothing to report.

## Supporting information


**Table S1:** Regional tariff nomenclature for outpatient specialist care, Emilia‐Romagna Region, Italy.

**Table S2:** Unit tariffs of the surveillance basket across health systems, and the resources released by the discontinuation rule under each.

**Table S3:** Sensitivity analyses of the resources released by discontinuation of surveillance.

**Table S4:** Risk of a new worrisome feature or high‐risk stigmata after a documented period of stability.

**Table S5:** Sensitivity analyses of the resources released.

## Data Availability

The data that support the findings of this study are available on request from the corresponding author. The data are not publicly available due to privacy or ethical restrictions.
