# Supplementary material for: Discontinuing Surveillance of Low‐Risk Branch‐Duct Intraductal Papillary Mucinous Neoplasms in Routine Practice: An External Application of the “Trivial” Criteria and the Resources it Would Release in Fourteen Health Systems
Source: United European Gastroenterol J. 2026 Oct 9;14(8):e70304. doi: 10.1002/ueg2.70304 (PMC13651003; doi:10.1002/ueg2.70304)
Supplement: Supplementary file 1 [file UEG2-14-e70304-s001.docx]

**Supplementary Table 3. Sensitivity analyses of the resources released by discontinuation of surveillance. All figures are recomputed on the basket of the base case, which includes the specialist review and the serum CA 19-9 determination accompanying each cross-sectional examination. Discounting, the contribution of endoscopic ultrasonography, protocol adherence in each health system and the composition of the basket are reported in Supplementary Table 5.**

| **Scenario** | **Patients**  **discharged,**  **n** | **Person-**  **years**  **avoided** | **Procedures**  **avoided, n** | **Cost avoided, €**  **(95% CI)** | **Per 1000 entering**  **surveillance, €** | **Events**  **not**  **detected** | **High-grade**  **lesions**  **missed** |
| --- | --- | --- | --- | --- | --- | --- | --- |
| ***Primary analysis*** |  |  |  |  |  |  |  |
| **Landmark 60 months, horizon 10 years, marginal cost** | **101** | **298** | **113** | **31 092 (16 450–46 972)** | **79 316** | 5 | **0** |
| ***Duration of stability required*** |  |  |  |  |  |  |  |
| 36 months | 153 | 494 | 204 | 56 504 (40 967–73 356) | **144 142** | 10 | **0** |
| 48 months | 125 | 388 | 155 | 43 131 (28 528–58 942) | **110 028** | 6 | **0** |
| 84 months | 80 | 156 | 60 | 16 392 (6 841–29 109) | **41 816** | 2 | **0** |
| ***Time horizon*** |  |  |  |  |  |  |  |
| 8 years from diagnosis | 101 | 228 | 86 | 23 797 (12 590–35 952) | **60 707** | 5 | **0** |
| 15 years from diagnosis | 101 | 335 | 126 | 34 911 (18 470–52 741) | **89 058** | 5 | **0** |
| Entire observed follow-up | 101 | 335 | 126 | 34 947 (18 489–52 796) | **89 150** | 5 | **0** |
| ***Cost basis*** |  |  |  |  |  |  |  |
| Average instead of marginal cost per person-year | 101 | 298 | 113 | 53 693 (49 961–57 566) | **136 972** | 5 | **0** |
| Pre-2024 tariff schedule | 101 | 298 | 113 | 30 776 (16 335–46 602) | **78 509** | 5 | **0** |
| ***Surveillance intensity*** |  |  |  |  |  |  |  |
| If surveillance continued at the average rate of the cohort | 101 | 298 | 172 | 47 309 (—) | **120 686** | 5 | **0** |
| If surveillance continued at the rate the protocol expects | 101 | 298 | 270 | 74 193 (—) | **189 268** | 5 | **0** |
| ***Cohort definition*** |  |  |  |  |  |  |  |
| Including ultrasound-only baseline work-up (n = 401) | 102 | 300 | 103 | 28 550 (15 630–43 541) | **71 198** | 5 | **0** |
| Including surveillance < 12 months (n = 424) | 101 | 298 | 113 | 31 092 (16 450–46 972) | **73 330** | 5 | **0** |
| Restricted to patients with CA 19-9 measured (n = 221) | 50 | 143 | 44 | 12 154 (5 039–20 698) | **54 997** | 4 | **0** |
| ***Probabilistic analysis*** |  |  |  |  |  |  |  |
| Monte Carlo, 50 000 simulations (median, 95% credible interval) | 101 | 761 | — | 79 286 (42 392–125 162) | **79 286** | — | **1.3** |
| Monte Carlo, health system drawn at random from the fourteen | 101 | 761 | — | 67 489 (30 160–213 119) | **67 489** | — | **1.3** |

**Legend:** All scenarios apply the combined discontinuation rule (age ≥ 75 years with a stable cyst < 30 mm, or age ≥ 65 years with a stable cyst ≤ 15 mm). The primary analysis is shaded; **Duration of stability.** The requirement for stability before discontinuation is the dominant determinant of the resources released: halving it from 60 to 36 months roughly doubles the saving, from €79 316 to €144 142 per 1000 patients entering surveillance. No high-grade dysplasia or invasive carcinoma would have been missed at any landmark, including 24 and 36 months. The single patient in whom high-grade dysplasia was found after two years of stability was 63 years old with a cyst of 18 mm and therefore met neither discontinuation rule, so the rule itself, and not only the waiting time, contributed to safety; **Time horizon.** The horizon determines for how long surveillance is assumed to continue in the absence of a discontinuation rule. Extending it from the ten-year base case to the entire observed follow-up raises the saving from €79 316 to €89 150 per 1000 patients, an increase of 12%; shortening it to eight years reduces it to €60 707; **Cost basis.** Valuing the released person-years at the average rather than the marginal cost per person-year nearly doubles the estimated saving, from €79 316 to €136 972 per 1000 patients, and would overstate it, because examinations are concentrated in the early years of surveillance. Applying the tariff schedule in force before 2024 leaves the estimate essentially unchanged, because few endoscopic procedures are performed after the fifth year. Variation in unit costs across health systems is far larger than any of the alternatives explored here and is examined separately in Figure 3; **Surveillance intensity.** The marginal use of imaging beyond the fifth year was 0.38 examinations per person-year, substantially lower than the average rate of 0.57 over the whole period, because surveillance de-escalates as it lengthens. Only 67% of the cross-sectional examinations expected under the local protocol were actually performed. Two counterfactuals are shown: continuation at the cohort’s own average rate, and at the rate the protocol expects. Neither is the primary estimate, because a payer can only release money it is actually spending; both indicate what the same rule would release where magnetic resonance capacity and attendance are not constraints; **Probabilistic analysis.** A Monte Carlo model with 50 000 simulations propagated, jointly, the proportion of patients eligible for discontinuation (conjugate beta posterior), the person-years released per eligible patient (bootstrap), the marginal cost per person-year (bootstrap of the regression slope) and the rate of undetected high-grade lesions (Jeffreys gamma posterior for a Poisson rate with no observed events in 298.2 person-years of released exposure). The rate of undetected high-grade lesions was estimated from the exposure of the patients whom the rule would actually discharge (no event in 298 person-years); borrowing the exposure of all patients stable at five years (593 person-years) halves it and is reported as a sensitivity. The median saving reproduces the deterministic estimate. No high-grade lesion was missed in 54% of simulations (66% under the wider exposure) and at most one in 73%; the expected number was 1.3 per 1000 patients entering surveillance, against the €79 316 released; **Cohort definition.** Reinstating the patients whose baseline work-up consisted of transabdominal ultrasonography alone, or those under surveillance for less than 12 months, changes the estimate by about a tenth. Restricting the cohort to the patients in whom serum CA 19-9 had actually been measured reduces the number eligible for discontinuation by nearly half and the saving accordingly, but leaves the conclusion unaltered.
