# Supplementary material for: Discontinuing Surveillance of Low‐Risk Branch‐Duct Intraductal Papillary Mucinous Neoplasms in Routine Practice: An External Application of the “Trivial” Criteria and the Resources it Would Release in Fourteen Health Systems
Source: United European Gastroenterol J. 2026 Oct 9;14(8):e70304. doi: 10.1002/ueg2.70304 (PMC13651003; doi:10.1002/ueg2.70304)
Supplement: Supplementary file 2 [file UEG2-14-e70304-s002.docx]

**Supplementary Table 4.** Risk of a new worrisome feature or high-risk stigmata after a documented period of stability.

| **Duration of**  **stability** | **Patients still**  **stable, n (%)** | **Conditional 5-year risk**  **of a new WF/HRS,**  **% (95% CI)** | **Landmark 5-year risk**  **of a new WF/HRS,**  **% (95% CI)** | **Events per**  **100 person-**  **years** | **Resection /**  **high-grade**  **lesion, n** |
| --- | --- | --- | --- | --- | --- |
| **24 months** | 335 (85.5) | 14.3 (9.9–19.0) | 14.3 (9.7–18.8) | 4.4 | 1 / 1 |
| **36 months** | 294 (75.0) | 13.8 (9.4–18.9) | 13.8 (9.0–18.6) | 4.7 | 1 / 1 |
| **48 months** | 239 (61.0) | 11.1 (6.2–16.6) | 11.1 (5.9–16.3) | 3.8 | 0 / 0 |
| **60 months** | 195 (49.7) | 12.5 (6.6–19.1) | 12.5 (6.4–18.7) | 4.3 | 0 / 0 |

**Legend:** Cohort of 392 patients with low-risk branch-duct intraductal papillary mucinous neoplasm, of whom 66 developed a new worrisome feature or high-risk stigmata during surveillance; **Conditional risk.** Probability of developing a new worrisome feature or high-risk stigmata within the following five years, given that no such feature had appeared up to the stated duration of stability, estimated as 1 − S(t+60)/S(t) from the Kaplan–Meier estimate of the cohort. Confidence intervals were obtained by bootstrap resampling with 2000 replicates; **Landmark analysis.** Kaplan–Meier estimate restricted to patients who reached the landmark free of events, with time reset to zero at that point. The two columns are algebraically equivalent, because the ratio S(t+60)/S(t) depends only on the events occurring after the landmark and therefore on the same patients; their agreement confirms the computation rather than adding information. Both are estimated from the patients whose observation extended beyond the landmark, whose number is given in the second column; **Resection and high-grade lesion.** Number of patients still stable at the landmark who subsequently underwent pancreatic resection, and number in whom high-grade dysplasia or invasive carcinoma was found. No resection and no high-grade lesion occurred after four years of documented stability. WF, worrisome feature; HRS, high-risk stigmata; CI, confidence interval.
