# Supplementary material for: Discontinuing Surveillance of Low‐Risk Branch‐Duct Intraductal Papillary Mucinous Neoplasms in Routine Practice: An External Application of the “Trivial” Criteria and the Resources it Would Release in Fourteen Health Systems
Source: United European Gastroenterol J. 2026 Oct 9;14(8):e70304. doi: 10.1002/ueg2.70304 (PMC13651003; doi:10.1002/ueg2.70304)
Supplement: Supplementary file 3 [file UEG2-14-e70304-s003.docx]

**Supplementary Table 2.** Unit tariffs of the surveillance basket across health systems, and the resources released by the discontinuation rule under each.

*Unit tariffs are shown in the currency of the health system; the cost per person-year applies the resource use observed in the study cohort (0.5381 magnetic resonance and 0.0994 computed tomography examinations per person-year) and is converted to euro at European Central Bank reference rates of 21 August 2026. The study setting is shaded blue; rows shaded amber carry a qualification given in the note.*

| **Health system** | **Note** | **Magnetic**  **resonance** | **Computed**  **tomography** | **Currency** | **Cost per**  **person-year, €** | **Relative**  **to study** | **Per 1000**  **entering, €** |
| --- | --- | --- | --- | --- | --- | --- | --- |
| Japan | 1 | 23 250.00 | 22 450.00 | JPY | **79** | ×0.54 | **42 485** |
| Spain | 2 | 148.00 | 120.00 | EUR | **92** | ×0.62 | **48 991** |
| France | 3 | 154.69 | 97.03 | EUR | **93** | ×0.63 | **49 696** |
| Romania | 4 | 850.00 | 469.00 | RON | **96** | ×0.65 | **51 302** |
| Belgium | 5 | 166.54 | 164.89 | EUR | **106** | ×0.72 | **56 717** |
| Germany | 6 | 182.57 | 92.24 | EUR | **107** | ×0.72 | **57 468** |
| Austria | 7 | 178.06 | 119.50 | EUR | **108** | ×0.73 | **57 619** |
| England — NHS | 8 | 188.00 | 98.00 | GBP | **129** | ×0.87 | **69 263** |
| **Italy — study institution** | 9 | 249.45 | 141.00 | EUR | **148** | ×1.00 | **79 316** |
| Poland | 10 | 1 133.92 | 630.80 | PLN | **156** | ×1.05 | **83 571** |
| United States — Medicare | 11 | 336.01 | 300.27 | USD | **180** | ×1.21 | **96 339** |
| Switzerland | 12 | 390.49 | 324.35 | CHF | **259** | ×1.75 | **138 642** |
| United States — commercial | 13 | 444–702 | 396–628 | USD | **307** | ×2.07 | **164 258** |
| Sweden | 14 | 5 905.00 | 3 468.00 | SEK | **318** | ×2.15 | **170 350** |

**Sources and qualifications**

1. Japan — 診療報酬点数表 FY2026: E202 1.5T + contrast + E203 + management; E200; D215

2. Spain — Andalusia: Orden 23-II-2023, BOJA 41/2023, tarifas a precio cerrado. Health care is devolved to the autonomous communities; no national schedule exists

3. France — CCAM act + forfait technique (1.5T amortised, province), 1 July 2026

4. Romania — CNAS, tarife investigaţii paraclinice 2025 (cod 122, 76, 32)

5. Belgium — RIZIV/INAMI art. 17, honoraria 01/01/2026 (459410, 459572, 459712)

6. Germany — EBM 2026: 34440+34452, 34341, 33042; Orientierungswert 12.7404 ct

7. Austria — BVAEB MRT/CT Gesamtvertrag flat per case; ÖGK Anlage D Pos. 931

8. England — NHS — NHS Payment Scheme national prices 2025/26, unbundled subchapter RD

9. Italy — study institution — Institutional tariffs, Emilia-Romagna regional schedule (reference case). Italian tariffs are set regionally within a national ceiling; no single national value applies

10. Poland — NFZ AOS/ASDK points x 1.52 PLN (AOTMiT Rek. 72/2025)

11. United States — Medicare — CMS Physician Fee Schedule CY2026 national: 74183, 74177, 76700

12. Switzerland — TARMED positions x TPW 0.89; excludes Wechselzeit, Befundung and RIS/PACS positions, therefore an underestimate

13. United States — commercial — Commercial-to-Medicare multiplier 1.32–2.09 (observed 74183 allowed-amount spread; RAND Round 5.1 professional services 184%)

14. Sweden — Regional radiology price list 2026 (inter-regional billing price, includes overhead — not a national tariff)

**Health systems for which no comparable unit price could be established**

Endoscopic ultrasonography is excluded from this comparison. A separately priced diagnostic biliopancreatic or upper gastrointestinal examination could be identified in ten of the fourteen schedules and is absent from five; where it exists the amounts are not commensurable, some being professional fees with the facility cost met separately, others all-in episode prices, and in Japan a surcharge on the base endoscopy. Supplementary Table 5 gives the full comparison. Netherlands: imaging tariffs are freely negotiated between insurer and provider; NZa sets no unit tariff (BR/REG-24102a). Provider list prices for self-payers are €430 MRI, €260 CT, €190 ultrasound.

Denmark, Norway, Finland, Ireland: tax-funded systems with global budgets and no published per-examination price; only private self-pay lists are public.

Canada: provincial schedules pay a professional fee only for CT and MRI (Ontario X410 CAD 97.50); the technical cost sits in hospital global budgets and is not published.

Hungary, Czechia: procedure point counts are public but the monetary value of a point could not be verified from a primary source.

Greece, Bulgaria, Portugal: payer tariffs exist but were not retrievable in machine-readable form.

**Variation within Italy**

Since the Decree of 23 June 2023 the national tariff is a ceiling that regions may undercut. Most regions adopted it unchanged (Piedmont, Aosta Valley, Liguria, Tuscany, Lazio, Campania, Sicily, Bolzano); Lombardy, Sardinia, Veneto (from June 2025) and Emilia-Romagna set their own, and Abruzzo, under a deficit-recovery plan, explicitly applies the minimum values. The schedule of the study region, in force from 30 December 2024, prices magnetic resonance of the upper abdomen at €215.20 (88.95.2) with cholangiopancreatography at €138.10 (88.97.A), and computed tomography at €145.90 (88.01.2) and €181.70 (88.01.6); the corresponding national ceilings are €126.90 and €158.04. The imaging tariffs of the other regional schedules could not be extracted from the published documents, so internal Italian variation is described qualitatively and not quantified here.
