# Supplementary material for: Discontinuing Surveillance of Low‐Risk Branch‐Duct Intraductal Papillary Mucinous Neoplasms in Routine Practice: An External Application of the “Trivial” Criteria and the Resources it Would Release in Fourteen Health Systems
Source: United European Gastroenterol J. 2026 Oct 9;14(8):e70304. doi: 10.1002/ueg2.70304 (PMC13651003; doi:10.1002/ueg2.70304)
Supplement: Supplementary file 4 [file UEG2-14-e70304-s004.docx]

**Supplementary Table 5.** Sensitivity analyses of the resources released.

*All analyses apply the discontinuation rule to the combined criteria (101 of 392 patients, 298.2 person-years released). Unless stated otherwise, resources released are expressed in euro per 1000 patients entering surveillance, undiscounted, at the unit costs of the study setting, and the costed basket is that of the base case: cross-sectional imaging, endoscopic ultrasonography, and the specialist review and serum CA 19-9 determination that accompany each cross-sectional examination.*

**A. Discount rate (ten-year horizon)**

| **Annual discount rate** | **Person-years** | **Resources released, €** | **Per 1000 entering, €** |
| --- | --- | --- | --- |
| 0.0% (base case) | 298.2 | 31,092 | 79,316 |
| 3.0% | 282.0 | 29,394 | 74,985 |
| 3.5% | 279.4 | 29,129 | 74,309 |
| 5.0% | 272.1 | 28,363 | 72,355 |

**Legend:** Costs are discounted to the landmark at five years, the point at which the decision to discontinue is taken. The effect is small because the resources released accrue within a narrow window between the fifth and the tenth year. The rates recommended by the national agencies of the systems priced here (3.5% NICE, 3% IQWiG and US Public Health Service, 4% on costs in the Netherlands) all fall inside the range shown.

**B. Time horizon**

| **Horizon from diagnosis** | **Person-years** | **Per 1000 entering, €** | **Ratio to base** |
| --- | --- | --- | --- |
| Seven years | 169.6 | 45,113 | 0.57 |
| Ten years (base case) | 298.2 | 79,316 | 1.00 |
| Fifteen years | 334.9 | 89,058 | 1.12 |
| Twenty years | 335.2 | 89,150 | 1.12 |
| To the end of observed follow-up | 335.2 | 89,150 | 1.12 |

**Legend:** Ten years was chosen so that the estimate remains within the period the cohort actually contributes. Of the 101 patients the rule would discharge, 20 were followed beyond ten years and 1 beyond fifteen, the longest follow-up among them being 15.3 years. The cap is therefore conservative: removing it raises the estimate by 12%, and nothing further accrues beyond fifteen years because no patient contributes.

**C. Contribution of endoscopic ultrasonography in the study cohort**

| **Quantity** | **Value** | **Share** |
| --- | --- | --- |
| Examinations performed during surveillance, n | 56 | — |
| of which with tissue acquisition | 25 | — |
| Patients undergoing at least one, n | 49 | 12.5% |
| Examinations per person-year | 0.0236 | — |
| Cost per person-year, € | 9.49 | 5.3% |
| Marginal cost beyond the fifth year, €/person-year | 3.97 | 3.8% |
| Rate in patients who never developed a feature, /person-year | 0.0177 | — |
| Rate in patients who developed a feature, /person-year | 0.0482 | — |

**Legend:** Endoscopic ultrasonography accounts for 5.3% of the cost of surveillance and for 3.8% of its marginal cost beyond the fifth year. Endoscopic ultrasonography is performed on indication rather than on a fixed schedule: at the appearance of a new worrisome feature or high-risk stigma, or to characterize and follow a lesion already assessed endoscopically at diagnosis. The registry is consistent with this. Of the 56 examinations recorded during surveillance, 22 were performed in the 66 patients who developed a new feature — a rate 2.7 times higher per person-year than in those who did not — and a further 24 in patients who had already undergone endoscopic ultrasonography at baseline; the remaining 10, in 7 patients, fall outside both groups.

**D. Endoscopic ultrasonography in the fourteen payer schedules**

| **Health system** | **Code** | **Amount** | **€** | **What the price covers** | **Evidence** |
| --- | --- | --- | --- | --- | --- |
| **Italy — study institution** | **52.13** | **253.25 EUR** | **253** | **all-in outpatient tariff** | **Verified** |
| France | HMQJ001 | 251.46 EUR | 251 | professional fee only | Secondary |
| Belgium | 473852 | 331.56 EUR | 332 | physician honorarium only | Verified |
| Switzerland | VA.25.0010 | 341.87 CHF | 366 | professional and technical | Derived |
| England — NHS | FE22Z | 423.00 GBP | 494 | whole episode, no EUS-specific code | Secondary |
| Spain — Galicia | named line | 627.53 EUR | 628 | all-in closed tariff | Verified |
| United States — Medicare | CPT 43259 | 198.74 USD | 170 | professional only; facility paid separately | Secondary |
| Netherlands | 034388 | 1 097–1 266 EUR | 1,097 | integral tariff, set by each hospital | Verified |
| Sweden — Sydöstra region | 485-2-50012 | 64 365 SEK | 5,818 | all-in inter-regional episode price | Verified |
| Japan | D308 + surcharge | 14 400 JPY | 78 | no standalone code; 300-point surcharge on the base endoscopy | Verified |
| Spain — Andalusia | — | — | — | no endoscopic ultrasound line in the schedule | Absent |
| Germany | — | — | — | no endosonography position in the EBM | Absent |
| Austria | — | — | — | no position in the ÖGK or BVAEB schedules | Absent |
| Poland | — | — | — | only anorectal endosonography is listed | Absent |
| Romania | — | — | — | no entry in the CNAS paraclinical package | Absent |

**Legend:** A separately priced diagnostic biliopancreatic or upper gastrointestinal endoscopic ultrasound could be identified in ten of the fourteen systems and is absent from five of the schedules examined. Where it exists the amounts are not commensurable: France, Belgium and United States Medicare pay a professional fee with the facility cost met separately; Italy, Galicia, England, the Netherlands and Sweden set an all-in price for the episode; Switzerland combines a professional and a technical component; and Japan has no standalone code at all, pricing the examination as a 300-point surcharge on the base endoscopy. In England no endoscopic ultrasound health resource group exists, so the examination is paid at the generic diagnostic upper gastrointestinal endoscopy price; in the Netherlands the product exists but carries no maximum tariff and is priced by each hospital, the same code varying by 15% between three hospitals in the same year. Expressed in euro the range is 75-fold, and it is governed by what each price includes rather than by what the examination costs, so a like-for-like sensitivity analysis is not available. Where a national price does not exist, the schedule used for the endoscopic examination is not always the one used for imaging in Supplementary Table 2: the Swedish endoscopy price is the Sydöstra region’s inter-regional list and the Spanish one is Galicia’s, whereas the imaging tariffs are those of Region Halland and of Andalusia. Relative to magnetic resonance, endoscopic ultrasonography is priced higher in most of these systems than in ours (1.0 times the magnetic resonance tariff here, against 1.6 in France, 2.0 in Belgium and 2.3 in England), so including it would widen the cross-system spread rather than narrow it, and its exclusion is conservative with respect to our conclusion.

**E. Resources released by health system, at observed and at full protocol adherence**

| **Health system** | **At observed adherence, €** | **At full adherence, €** |
| --- | --- | --- |
| Japan | 42,485 | 101,379 |
| Spain — Andalusia | 48,991 | 116,906 |
| France | 49,696 | 118,587 |
| Romania | 51,302 | 122,420 |
| Belgium | 56,717 | 135,340 |
| Germany | 57,468 | 137,133 |
| Austria | 57,619 | 137,494 |
| England — NHS | 69,263 | 165,279 |
| Italy — study institution | 79,316 | 189,268 |
| Poland | 83,571 | 199,421 |
| United States — Medicare | 96,339 | 229,890 |
| Switzerland | 138,642 | 330,835 |
| United States — commercial | 164,258 | 391,962 |
| Sweden — Region Halland | 170,350 | 406,499 |

**Legend:** Our patients received 0.377 cross-sectional examinations per person-year beyond the fifth year against the 0.900 the institutional protocol prescribes, a ratio of 0.42. Actual-to-intended imaging ratios are not published for the other health systems and could not be constructed, so both bounds are given for every system: the left column applies the adherence observed in this cohort, the right assumes that every protocol-intended examination is performed. The two differ by a factor of 2.39 in every system, because adherence and tariff act multiplicatively on the same quantity, and the value for any given system lies between them. The cross-system comparison prices cross-sectional imaging only.

**F. Composition of the costed basket**

| **Basket** | **Visit, €** | **CA 19-9, €** | **Marginal cost, €/py** | **Per 1000 entering, €** |
| --- | --- | --- | --- | --- |
| Imaging and endoscopy only | — | — | 91.38 | 69,523 |
| + specialist review | 18.00 | — | 98.00 | 74,560 |
| + review and CA 19-9 | 18.00 | 17.00 | 104.25 | 79,316 |
| + review at the first-visit tariff and CA 19-9 | 25.00 | 17.00 | 106.83 | 81,275 |

**Legend:** Each cross-sectional examination is performed in the context of one specialist review, with a serum CA 19-9 determination requested alongside it, so visits and determinations enter the basket in the same number as the cross-sectional examinations. They are priced at codes 89.01.9 (visita gastroenterologica di controllo, €18.00) and 90.55.3 (antigene carboidratico 19.9, €17.00) of the same regional schedule. Their inclusion raises the marginal cost from €91.38 to €104.25 per person-year and the resources released from €69,523 to €79,316 per 1000 patients entering surveillance. Patient time, travel and lost productivity remain outside the analysis, which takes the perspective of the payer.

**G. Unit costs of the current regional schedule**

| **Unit costs applied** | **Marginal cost, €/py** | **Average, €/py** | **Per 1000 entering, €** |
| --- | --- | --- | --- |
| Institutional database, study period (base case) | **104.25** | **180.03** | **79,316** |
| Regional schedule in force from 30 December 2024 | 94.65 | 165.65 | 72,008 |

**Legend:** The costs analyzed were incurred between 2012 and 2024 and are valued at the unit prices the institutional administrative database applied over that period. The regional schedule that took effect on 30 December 2024, at the very end of the study period, prices magnetic resonance of the upper abdomen at €215.20 and computed tomography of the whole abdomen at €181.70, against the €249.45 and €141.00 applied historically; the endoscopic ultrasonography codes are unchanged. The direction of the change differs between modalities, so the two sets are not a simple uplift of one another.

**H. The prior for a rate with no observed events, and the inputs of the probabilistic model**

| **Quantity** | **Distribution** | **Source** |
| --- | --- | --- |
| Proportion eligible for discontinuation | Beta(101, 291) | conjugate posterior on the observed 101 of 392 |
| Person-years released per eligible patient | non-parametric bootstrap | observed per-patient values, 2,000 replicates |
| Marginal cost per person-year | bootstrap of the regression slope | cost on duration, patients followed beyond five years |
| Rate of undetected high-grade lesions | Gamma(0.5, 298.2) | Jeffreys posterior, no event in 298.2 person-years |
| Lesions not detected | Poisson at the sampled rate and exposure | — |

**Legend:** For a Poisson rate λ observed over T person-years with x events, the Jeffreys prior is proportional to λ raised to the power −½, which is the square root of the Fisher information and is therefore invariant to reparameterization: the same prior is obtained whether the quantity of interest is the rate, its logarithm or its reciprocal. The posterior is Gamma(x + ½, T). With x = 0 and T = 298.2 person-years released by the combined rule, the posterior median is 0.074 and the 97.5th percentile 0.84 events per 100 person-years. Unlike the rule of three, which returns a single upper bound and is undefined as a distribution, this yields the whole posterior, so it can be propagated through the probabilistic model and can answer the question a reader actually asks, namely how often no lesion at all would be missed.Fifty thousand simulations were drawn jointly. The median resources released, €79,286 per 1000 patients entering surveillance, reproduces the deterministic estimate of €79,316, and the 95% credible interval is €42,392 to €125,162. No lesion escaped detection in 54% of simulations and at most one in 73%; borrowing the exposure of all patients stable at five years (593 person-years) raises these to 66% and 85% and is reported as a sensitivity. The seed and the code are provided with the analysis package.
