# Supplementary material for: Discontinuing Surveillance of Low‐Risk Branch‐Duct Intraductal Papillary Mucinous Neoplasms in Routine Practice: An External Application of the “Trivial” Criteria and the Resources it Would Release in Fourteen Health Systems
Source: United European Gastroenterol J. 2026 Oct 9;14(8):e70304. doi: 10.1002/ueg2.70304 (PMC13651003; doi:10.1002/ueg2.70304)
Supplement: Supplementary file 5 [file UEG2-14-e70304-s005.docx]

**Supplementary Table 1. Regional tariff nomenclature for outpatient specialist care, Emilia-Romagna Region, Italy. Unit costs are the values applied in the institutional administrative database over the study period.**

| **Code** | **Procedure** | **Unit cost (€)*** |
| --- | --- | --- |
| **88.95.2^** | Magnetic resonance imaging of the upper abdomen, without and with contrast, including magnetic resonance cholangiopancreatography | 249.45 |
| **88.01.6** | Computed tomography of the whole abdomen, without and with contrast | 141.00 |
| **52.13°** | Biliopancreatic endoscopic ultrasonography | 253.25 |
| **52.14** | Transparietal (endoscopic ultrasound-guided) biliopancreatic biopsy | 556.80 |
|  | **Total** |  |

**Legend: *=** Available at <https://salute.regione.emilia-romagna.it/ssr/strumenti-e-informazioni/nomenclatore-tariffario-rer>; ^= Surveillance magnetic resonance examinations comprised imaging of the upper abdomen with cholangiopancreatography; °= Under the regional nomenclature, codes 52.13 and 52.14 are not mutually associable: an endoscopic ultrasound performed with tissue acquisition is billed as 52.14 and replaces, rather than adds to, 52.13. Procedures were therefore attributed to code 52.14 when a cytological or histological report was available and to code 52.13 otherwise. Three patients had a cytological or histological report without a separately recorded endoscopic ultrasound procedure and were costed under code 52.14. Each cross-sectional examination is accompanied by one specialist review and one serum CA 19-9 determination, coded 89.01.9 (visita gastroenterologica di controllo, €18.00) and 90.55.3 (antigene carboidratico 19.9, €17.00) in the same schedule; both are included in the costed basket. In the regional schedule in force from 30 December 2024 the imaging values are €215.20 for code 88.95.2 and €138.10 for code 88.97.A, €181.70 for code 88.01.6 and €145.90 for code 88.01.2, while codes 52.13 and 52.14 are unchanged; costing the same resource use at that schedule is reported in Supplementary Table 5.
